# Sickle Cell Disease: Thoughts for India From the Jamaican Cohort Study

**DOI:** 10.3389/fmed.2021.745189

**Published:** 2021-11-05

**Authors:** Graham R. Serjeant

**Affiliations:** Sickle Cell Trust, Kingston, Jamaica

**Keywords:** sickle cell disease, Jamaica, cohort study, India, geographic comparison

## Abstract

The sickle cell gene in India represents a separate occurrence of the HbS mutation (the Asian haplotype), which has occurred against a genetic background characterised by high levels of fetal haemoglobin and widely varying frequencies of alpha thalassaemia. These features, which tend to inhibit sickling, change the expression of the disease, which, in India, may be further modified by poor nutrition, malaria and other infections, and limited public health resources. Sickle cell disease in Jamaica is predominantly of African origin (the Benin haplotype) and faces some similar challenges. This review assesses similarities and differences between disease expression in the two countries and seeks to explore lessons from Jamaica, which may be relevant to Indian health care. In particular, it addresses common causes of hospital admission as detailed from Indian clinical experience: anemia, bone pain crisis, and infections.

## Introduction

The sickle cell gene in India was first reported among tribal people in the Nilgiri Hills of Tamil Nadu in the south of the country (1). Since then, the distribution of HbS has been well documented across Gujarat, Maharashtra, Madhya Pradesh, Andhra Pradesh, Chhattisgarh, and western Odisha with a smaller focus in the southern states of Tamil Nadu and Kerala (2).

In peoples of African origin, there are three major beta globin haplotypes (Benin, Bantu, and Senegal), and the clinical features of the Benin haplotype, which dominates in North America and the Caribbean, have been well documented, but only limited information is available on the Asian haplotype, which accounts for the disease in India and the Eastern Province of Saudi Arabia. For brevity in the text, patients of African origin are referred to as African HbSS and those with the Asian haplotype as Asian HbSS. An early study of 131 patients with Asian HbSS from Burla Medical College in Western Odisha (3) found a disease relatively mild compared to Jamaica with little chronic leg ulceration and priapism, although hypersplenism appeared common. This impression of mildness was supported by the finding of 15 cases of unsuspected SS disease among parents and older relatives. Later studies from central India (4–6) reported a more severe clinical course but the extent to which these differences are influenced by varying ascertainment biases is uncertain. Newborn screening should avoid a symptomatic bias but only if complete follow-up is possible and two major programmes based on newborn screening in south Gujarat (7) and Nagpur in central India (6) were marred by default rates of 30%−36% within the first year. The current review seeks to clarify this rather confusing picture, assessing the evidence and, where possible, comparing Indian findings with those in the Jamaican Cohort Study (8). In particular, it focuses on the most common causes of hospital admission in Nagpur (4): anemia, bone pain crises, and infections.

## Anaemia

### The Haematology of HbSS in Jamaica and Odisha

**Figure 1:**
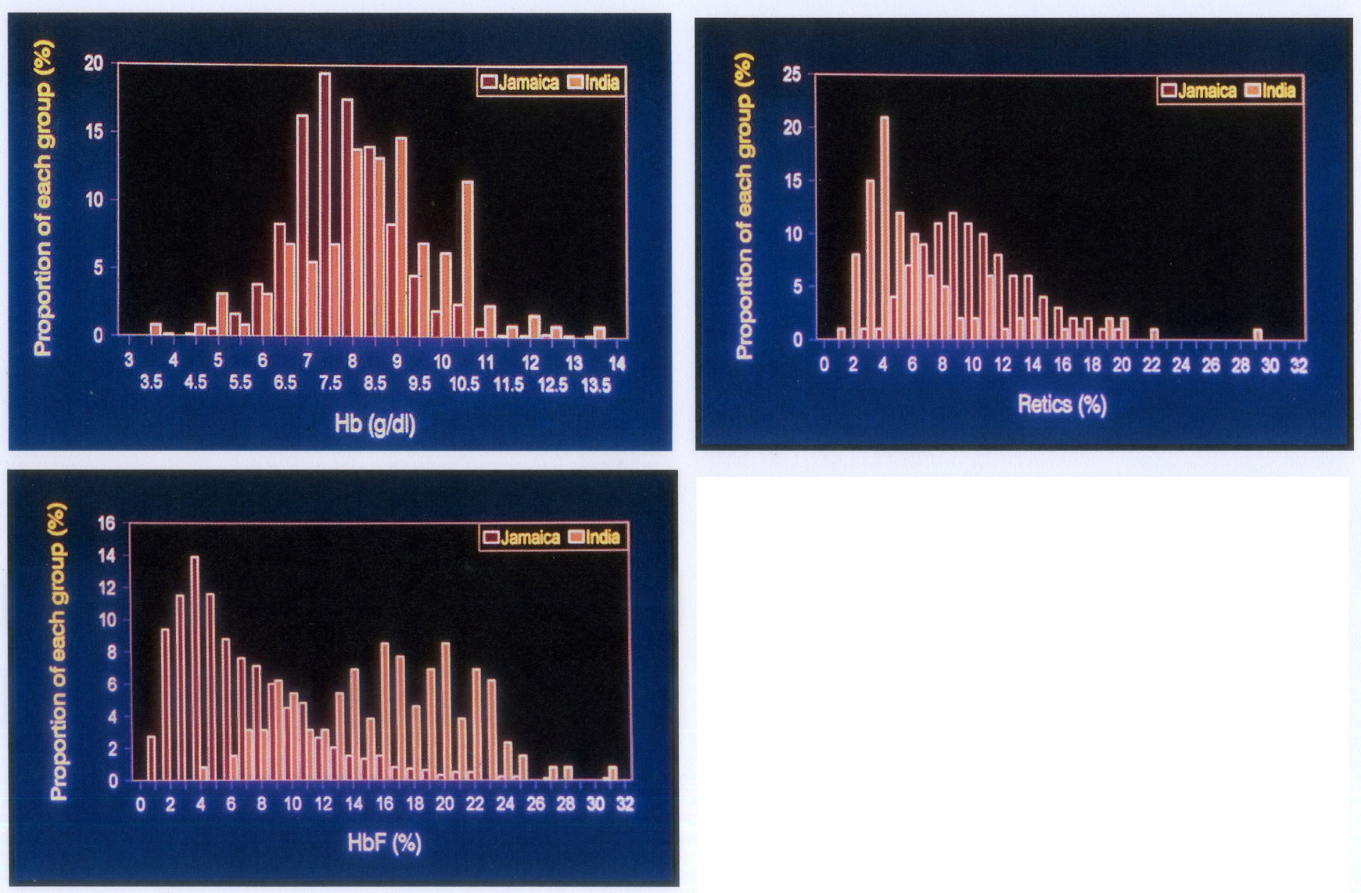
Comparison of haematological indices in SS disease of African origin (red columns) and Indian patients from Odish. **Top left:** haemoglobin levels, **Top right:** reticulocyte counts, **Lower left:** fetal haemoglobin levels. All differences are highly significant *p* < 0.001.

All patients with homozygous sickle cell (HbSS) disease have a shortened red cell survival due, in part, to the effect of sickle haemoglobin (HbS) in reducing the deformability of red cells. HbS within the red cell has a low oxygen affinity becoming nearly fully saturated in the lungs but releasing more oxygen per gram on haemoglobin in the periphery. Both these factors should be borne in mind when assessing haemoglobin levels in HbSS. In Jamaican patients, the mean red cell survival was 12.6 days, which ranged between 4.2 and 25.0 days (9) (compared to approximately 120 days in normal people). For comparison of Jamaica and Odisha, Jamaican data were derived from the non-Cohort clinic of 1,389 subjects, since this population provided more appropriate age matching with Odisha patients (3). The results showed that Odisha patients had significantly higher haemoglobin levels (mean 8.7 vs. 8.0 g/dl), lower reticulocyte counts (mean 6.5 vs. 10.5%), and much higher fetal haemoglobin (HbF) levels (16.6 vs. 6.1%) (Figure 1).

Molecular, haematological, and selected clinical features show many differences between Odisha and Jamaica (10).

### Anaemia Due to Impaired Bone Marrow Response

#### Aplastic Crisis

In Jamaica, acute anaemic events defined by haemoglobin levels of 3–4 g/dl and absent reticulocytes show seroconversion to parvovirus B19 in 95%, (11) and seroconversion in the cohort occurred in 60% of SS subjects by age 15 years (12). Parvovirus attacks red cell precursors in the bone marrow, and the development of parvovirus antibodies usually allows bone marrow recovery within 7–8 days, but this may exceed the mean red cell survival causing death in some patients with African HbSS. In Jamaica, treatment consists of outpatient transfusion of a single unit of blood with follow-up after 3–4 days to confirm the reticulocytosis of spontaneous bone marrow recovery. Parvovirus B19 is highly infectious and approximately 80% of susceptible siblings become aplastic either simultaneously or within 3 weeks so susceptible siblings should be closely monitored. The good news is that immunity to parvovirus appears to be lifelong and recurrence of parvovirus-induced aplasia has never been reported in SS disease.

In India, the frequent lack of reticulocyte counts in the investigation of acute anaemia does not allow a clinical definition of aplastic crisis, so comparable data are not available but the virus is common as seroconversion to parvovirus B19 occurred in 70% of healthy adult blood donors in India (13). Red cell survival in Asian HbSS is unknown but it would be expected that the commonly associated high levels of HbF may prolong red cell survival, diminishing the fall in haemoglobin level and the clinical severity of aplasia.

#### Hypoplasia From Nutritional Factors

##### Folate Deficiency

A falling haemoglobin associated with lower reticulocytes (1–4%) may reflect relative bone marrow hypoplasia from nutritional deficiency, infections, or metabolic problems such as renal failure. The mean cell volume (MCV) is useful in distinguishing iron deficiency (MCV low), or folate deficiency (MCV high), compared to the patients' steady-state values. The expanded bone marrow in SS disease increases the requirements for folic acid, and folate deficiency may lead to megaloblastic change with falling haemoglobin and increasing MCV. Observations from West Africa on the frequency of folate-deficient megaloblastic change (14) led to the widespread use of folate supplementation for SS disease elsewhere but the low dietary folate levels in countries such as Nigeria may render patients especially prone to this complication. The metabolic demands for folic acid are increased in SS disease but the crucial question is whether or not these are met by the diet.

In Jamaica, the dietary content of folic acid is relatively high and folate supplementation had little demonstrable effect on the clinical or haematological features of HbSS (15) so routine folate supplementation seems unnecessary although changing economic circumstances may influence policies (16). In India, the situation is largely unknown although dietary limitation for economic, religious, or cultural reasons may contribute to bone marrow hypoplasia. Folate deficiency, suspected on the basis of >3 lobes in neutrophils, occurred in over 80% of some populations in a National Multi-centric ICMR study of tribal populations (17).

##### Iron Deficiency

Although it is commonly assumed that iron from haemolysis is available for recycling in HbSS and that iron deficiency is unusual, the ICMR multi-centric study found evidence of iron deficiency in two-thirds of patients with Asian HbSS and mean haemoglobin levels increased by 2 g/dl after 3 months of iron supplementation (18).

##### Hypoplasia From Renal Failure

In Jamaica, renal function declines in most SS patients beyond the age of 40 years (19) with an associated fall in total haemoglobin (20) presumed to be consequent on reduced erythropoietin. Such patients are treated by top-up transfusions indicated by patient symptoms, and it is remarkable how low haemoglobin levels are tolerated by some elderly patients. In India, chronic renal impairment occurs in some elderly SS patients, but its prevalence and natural history are unknown.

### Red Cell Sequestration

Acute or chronic sequestration, most commonly in the spleen, may contribute to lower haemoglobin and higher reticulocyte counts.

#### Acute Splenic Sequestration (ASS)

In the Jamaican cohort, this was a major cause of early death, (21) and events characterised by acute splenic enlargement of 4 cm or more below the costal margin, a lowered haemoglobin level and increased reticulocytes, occurred in a quarter of children by 2 years of age. Most events resolved after blood transfusion and the spleen diminished in size. Recurrence was common, often at shorter intervals with continuing mortality leading to a policy of splenectomy after two attacks. Parents were taught to feel for the spleen every day when bathing their child and to contact the clinic immediately if they suspected enlargement. Analysis of 5 years before, and after, this education programme showed an apparent increased incidence from 4.6 to 11.3 per 100 patient/years, indicating events detected by the mother and confirmed by doctors at the clinic so families can be taught to detect this complication. The death rate fell from 29.4 to 3.1 per 100 events (22).

In India, this complication is known to occur (6), but its natural history and the role of splenectomy in its management are unknown.

#### Chronic Hypersplenism (CHS)

Sometimes splenomegaly is sustained with prolonged red cell sequestration and a new haematological equilibrium with haemoglobin levels (usually 4–6 g/dl), and higher reticulocyte counts (usually over 15%) and red cell survival may be as short as 1–3 days. The spleen is usually 4 cm or more below the costal margin and often much larger and the metabolic demands of the expanded bone marrow compete with those for growth and height velocity may fall (23). The essential difference between the pathology of ASS and CHS is currently unclear.

In the Jamaican cohort, this complication occurred in approximately 10% and cases are monitored at monthly intervals with haemoglobin and reticulocyte counts and measurement of height, and if there is no evidence of spontaneous resolution within 6 months or if height velocity crosses growth centiles, then splenectomy is performed. An alternative approach is chronic transfusion, but this has been found to be impractical under Jamaican conditions. Two Jamaican patients died from superimposed ASS while awaiting splenectomy for CHS.

**Figure 2:**
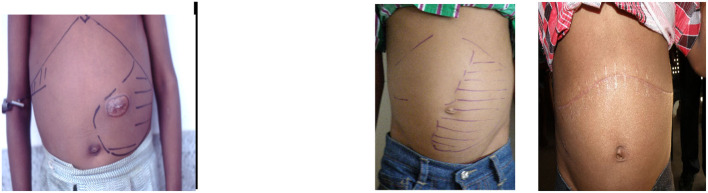
**Left**, 13-year-old patient with 12 cm spleen and splenic burn 4 months earlier. **Right**, 12-year-old patient from Valsad with 16 cm spleen and 3 months after splenectomy.

In India, CHS occurs and may be more common than in Jamaica. In the Odisha study, splenomegaly of 4 cm or more occurred in 33/122 (27%) patients but splenomegaly does not necessarily imply increased sequestration. Local awareness of an enlarged spleen may lead to branding over the splenic area (Figure 2), and others are treated by multiple transfusions. A 12-year-old patient attending the clinic in Valsad, Gujarat, had had 69 transfusions with a spleen measuring 16 cm below the left costal margin extending into the right iliac fossa (Figure 2), and following splenectomy, he has required no transfusions over the past 8 years. Splenectomy in the treatment of CHS has worked well in Jamaica, which is malaria free, but the role of malaria may influence the decision for splenectomy and requires independent assessment in India.

### Comment on Anaemia

Most patients with African HbSS, while in the steady state, maintain haemoglobin levels of 6–9 g/dl with reticulocyte counts of 8–12%, well below maximal bone marrow activity, and because HbS has a decreased oxygen affinity, oxygen delivery is near normal. When haemoglobin levels deviate from steady-state values characteristic of that patient, there is always a reason and, where possible, this should be investigated and addressed rather than the “blanket” treatment of blood transfusion. Indeed in some Indian clinics, patients are transfused at steady-state levels simply to raise their haemoglobin to what the doctor considers a “normal” level. Then, as the haemoglobin returns to the steady-state levels, they are transfused again because of the falling haemoglobin. This is unnecessary and poor practise exposing the patient to cost and potentially serious complications.

## Bone Pain

Avascular necrosis of the bone marrow results in a spectrum of clinical conditions, including dactylitis (hand-foot syndrome), bone pain crisis of adolescence and early adult life, and avascular necrosis of the femoral head (ANFH), each of which has a characteristic epidemiology.

### Dactylitis

**Figure 3:**
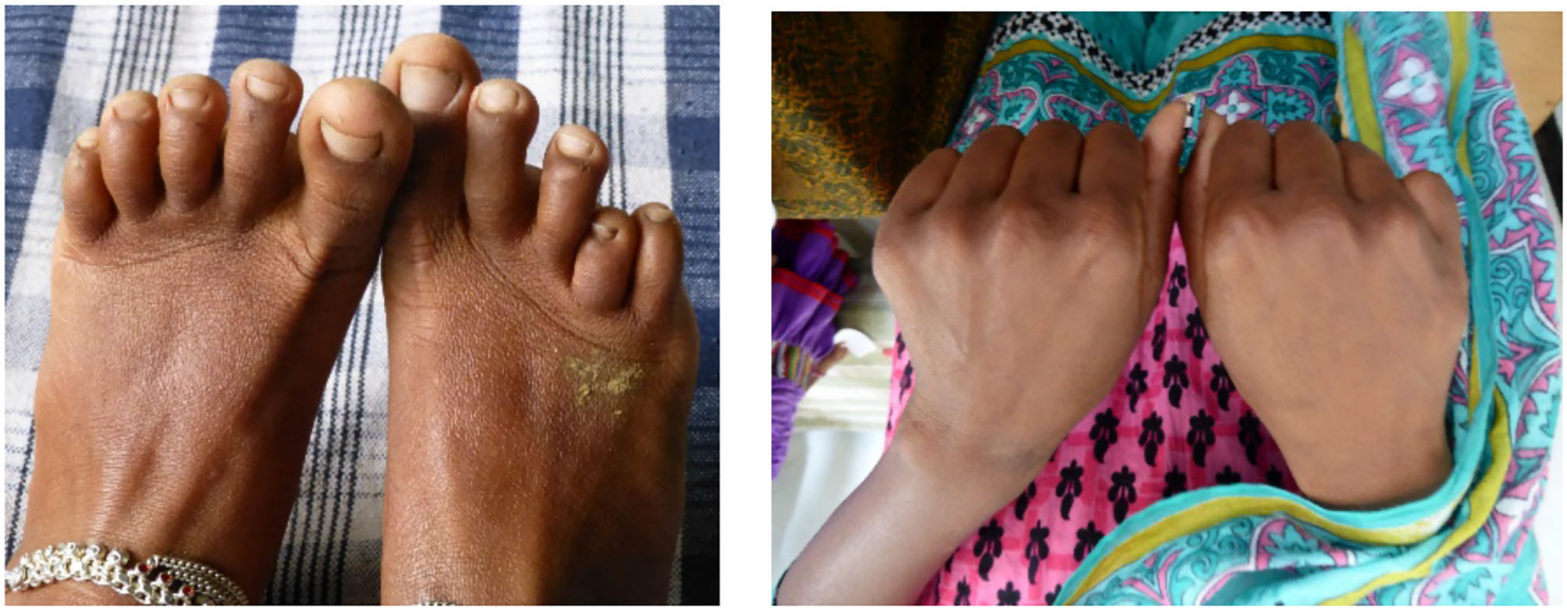
Subjects attending sickle cell camps in Chhattisgarh, India. **Left**, shortened fourth metatarsal of the right foot. **Right**, shortened fifth metacarpal of the right hand. Both almost certainly the sequel of infection superimposed on dactylitis.

In the Jamaican cohort, dactylitis commenced at 3–4 months of age, affected 45% by 2 years of age (24), and became rare after the age of 5 years when the susceptible bone marrow no longer occupies the small bones of the hands and feet. The only comparable Indian data derive from the cohort study in Nagpur in which dactylitis occurred in 9% by the age of 5 years (6), although a history of dactylitis was given in 52% in the Burla study (3). Dactylitis commonly recurs and usually resolves completely although transient bacteraemias may cause premature fusion and a permanent shortening of the affected small bones (Figure 3).

### Bone Pain Crisis

In Jamaica, the bone pain crisis affects principally the juxta-articular areas of the long bones, ribs, and spine commencing in later childhood, increasing in frequency during adolescence, especially in males and tapering in frequency and severity after the age of 30 years (25). Risk factors include a high total haemoglobin (26, 27) and low HbF (27), and common precipitating factors are skin cooling from exposure to cold water, baths, and rain. When associated with high fever or chest signs, hospital investigation may be necessary, but in their absence, this complication is essentially pathologically benign and home management is chosen in over 90% of cases admitted to the day-care centre in Jamaica (28) and elsewhere (29). Jamaica uses the WHO analgesic ladder commencing with paracetamol and paracetamol/codeine mixtures, the latter available without prescription and providing more adequate pain relief than paracetamol alone. Analgesic policy may vary between countries, and it is understood that narcotic agents cannot be given for home use in France, but in Jamaica, this has worked well.

In India, the limited data suggest a similar clinical pattern, and this complication is a common cause of hospital admission. However, only weak analgesics such as paracetamol are available for home use forcing patients requiring stronger pain relief to attend hospitals with the associated costs, difficulties, and disruption – indeed, the journey to hospital or a local clinic for stronger pain relief is only likely to increase anxiety and the degree of pain. Although some tablets combining paracetamol and codeine are available in India, these are not widely used and home analgesic policy for SS patients in India requires urgent revision.

### Avascular Necrosis of the Femoral Head

In Jamaica, this occurred in about 10% of the cohort usually developing in adolescence or early adult life. The outcome depends upon the age at which avascular necrosis occurs, involvement of the immature capital epiphysis often allowing retention of the joint space, and remoulding of the hip, whereas the involvement of the mature capital epiphysis causes secondary osteoarthritis with persistent pain and limitation of movement. Early diagnosis and avoidance of weight bearing may retard progression but eventually many patients require remodelling of the femoral head or total hip replacement.

In India, the prevalence is unknown but it is common, and requires study of its own risk factors, natural history, and protocols for optimal management.

## Infections

### Spectrum of Bacteria

In Jamaica, review of 93 initial septicaemic events in the cohort found 28 (30%) due to *Streptococcus pneumoniae*, 17 to *Salmonella*, 12 to *Staphylococci*, 11 to *Haemophilus*, 8 to *Klebsiella*, 6 to *Escherichia coli*, 5 to *Enterobacter*, and 6 others (Rankine-Mullings – unpublished).

### Streptococcus Pneumoniae

Invasive pneumococcal disease (IPD) affected approximately 10% by the age of 12 years in the Jamaican cohort (30), and pneumococcal prophylaxis has become routine (31, 32) in African HbSS. In India, paediatric studies from Nagpur found that febrile events were common causes of hospital admission (4, 6), and organisms isolated on culture included *Staphylococci, E. coli*, and *Klebsiella*, with a notable absence of *S. pneumoniae*.

In African HbSS, splenomegaly precedes the loss of splenic function, which was abnormal as early as the first year of life (33, 34). There is then an age-related decline in frequency of splenomegaly (35). In Asian HbSS, splenomegaly develops later and persists for longer (3, 36). Pitted red blood cells, which correlate with other indices of splenic function, such as Howell-Jolly bodies and technetium (^99m^Tc) sulphur colloid scans (37), increased later in Asian HbSS in eastern Saudi Arabia (38), and in western Odisha (3, 39), compared to Jamaica (40). Since the age specificity of IPD declines after the age of 3 years (41), persistence of splenic function beyond 3 years could explain the apparent absence of IPD in Indian experience and casts doubt on the need for pneumococcal prophylaxis.

### Salmonella

In the Jamaican cohort, the second most common organism was *Salmonella*. Although it is well known to be associated with osteomyelitis in HbSS, the absence of clinical bone involvement in a septicaemic child led to the assumption of IPD and antibiotic therapy inappropriate for *Salmonella* resulted in a mortality rate of 23% (42). Salmonella carriage is likely to be more common in India, and more extensive blood cultures are needed to define the spectrum of septicaemic disease in Indian patients.

### Other Infections

Malaria no longer exists in Jamaica but *Plasmodium vivax* has been shown to be common among febrile HbSS patients in Gujarat (43).

## Differences Between African HbSS and Asian HbSS

There are many similarities between African HbSS, as observed in Jamaica, and Asian HbSS but also some major differences. In the Jamaican cohort, chronic leg ulceration, defined by a minimal duration of 6 months, occurred in 30% (44) and priapism in 33% (45), but in Indian patients, both complications are rare and probably occur in less than 1%. The persistence of splenic function beyond the high-risk period for IPD may be vitally important casting doubt on the need for pneumococcal prophylaxis. A further difference relates to the use of hydroxyurea, which in African HbSS requires near toxic doses of 35 mg/kg requiring regular monitoring of haematology, whereas in Asian HbSS, a substantial elevation of HbF may be achieved by much smaller doses of 10 mg/kg (46, 47), implying less risk and less-intensive haematological monitoring.

A direct comparison between newborn cohorts in Jamaica and Nagpur, India, confirmed that Indian patients had higher levels of HbF, later appearance of splenomegaly, and absence of IPD (48).

### Comparison of HbSS Within India

Current claims of differences between HbSS in different parts of India must be treated with caution because of the different methods of patient ascertainment. The patients from Western Odisha (3, 10) represented survivors and also the chance event of awareness of the study. The supposedly more severe disease described in central India and south Gujarat is based on newborn screening but compromised by biases of selective default where failure to attend may be due to the lack of symptoms or death with their different implications. In India, HbF levels are consistently elevated compared to Jamaican patients but the prevalence of alpha thalassaemia defined by at least one alpha globin gene deletion varied widely in India, occurring in 16% in Akola (5), 28% in Nagpur (6), 53% in western Odisha (3), and 92% in South Gujarat (7) compared to 37% in Jamaica. This may contribute to possible regional clinical variation in India but the necessary clinical data are not yet available.

## Conclusions

This review has focused on the most common causes of hospital admission in children with Asian HbSS in Nagpur, India, and allowed some comparisons with Jamaican data. Haemolysis is increased in both populations but a lower haemolytic rate may occur in Indian disease consequent on the higher HbF levels. In both Jamaica and India, bone pain continues to be a major cause of hospital admission, and the wider use of stronger analgesics may allow more Indian patients to be treated at home. There is an impression that ANFH may be more common in India although this may reflect symptomatics selection. In Indian patients, splenic function persists beyond the high risk period for IPD with profound implications for clinical management. These differences also provide insights into the pathological mechanisms of the disease, which in India occurs against a genetic background of universally elevated HbF levels but variable frequencies of alpha thalassaemia. Some of the apparent variations between Jamaican and Indian disease may reflect social, cultural, and economic differences in the societies. Indian colleagues may learn from protocols developed for African disease, but these may not always be appropriate for Indian practise. Careful documentation of the disease in India, preferably by following cases diagnosed at birth, will be essential in evolving locally appropriate models of care.

## Author Contributions

GS has worked in Jamaica and India on sickle cell disease for many years and is the sole author of this review.

## Funding

The Jamaican Cohort Study was supported by the British Medical Research Council (MRC) through their support of the MRC Laboratories at the University of the West Indies. The MRC also covered the costs of transporting the laboratory equipment to India. The British Council covered the costs of airfares and subsistence for Prof. & Mrs. Serjeant during the study.

## Conflict of Interest

The author declares that the research was conducted in the absence of any commercial or financial relationships that could be construed as a potential conflict of interest.

## Publisher's Note

All claims expressed in this article are solely those of the authors and do not necessarily represent those of their affiliated organizations, or those of the publisher, the editors and the reviewers. Any product that may be evaluated in this article, or claim that may be made by its manufacturer, is not guaranteed or endorsed by the publisher.

## References

[1] Lehmann H Cutbush M. Sickle-cell trait in Southern India. Br Med J. (1952) 1:404–5. 10.1136/bmj.1.4755.404 14896162 PMC2022731

[2] Hockham C Bhatt S Colah R Mukherjee MB Penman BS Gupta S. The spatial epidemiology of sickle-cell anaemia in India. Sci Rep. (2018) 8:17685. 10.1038/s41598-018-36077-w 30523337 PMC6283872

[3] Kar BC Satapathy RK Kulozik AE Kulozik M Sirr S Serjeant BE. Sickle cell disease in Orissa State, India. Lancet. (1986) 328:1198–201. 10.1016/S0140-6736(86)92205-1 2430154

[4] Jain D Italia K Sarathi V Ghosh K Colah R. Sickle cell anemia from central India: a retrospective analysis. Indian Pediatr. (2012) 49:911–3. 10.1007/s13312-012-0217-z 22728629

[5] Jain D Warthe V Diyama P Sarate D Colah R Mehta P. Sickle cell disease in Central India: a potentially severe syndrome. Indian J Pediatr. (2016) 83:1071–6. 10.1007/s12098-016-2081-7 27053181

[6] Upadhye DS Jain DL Trivedi YL Nadkarni AH Ghosh K Colah RB. Neonatal screening and the clinical outcome in children with sickle cell disease in central India. PLoS ONE. (2016) 11:e0147081. 10.1371/journal.pone.0147081 26785407 PMC4718540

[7] Italia Y Krishnamurti L Mehta V Raicha B Italia K Mehta P. Feasibility of a newborn screening and follow-up programme for sickle cell disease among South Gujarat (India) tribal populations. J Med Screen. (2015) 22:1–7. 10.1177/0969141314557372 25341880

[8] Serjeant GR Serjeant BE Forbes M Hayes RJ Higgs DR Lehmann H. Haemoglobin gene frequencies in the Jamaican population: a study of 100,000 newborns. Br J Haematol. (1986) 64:253–62. 10.1111/j.1365-2141.1986.tb04117.x 3778823

[9] Serjeant G Serjeant B Stephens A Roper D Higgs D Beckford M. Determinants of haemoglobin level in steady-state homozygous sickle cell disease. Br J Haematol. (1996) 92:143–9. 10.1046/j.1365-2141.1996.284816.x 8562387

[10] Serjeant GR Kulozik AE Serjeant BE. Odisha revisited: a personal account. Front Med. (2022). 10.3389/fmed.2021.745337 PMC8581170 34778308

[11] Serjeant GR Topley JM Mason K Serjeant BE Pattison JR Jones SE. Outbreak of aplastic crises in sickle cell anaemia associated with parvovirus-like agent. Lancet. (1981) 2:595–7. 10.1016/S0140-6736(81)92739-2 6116082

[12] Serjeant BE Hambleton IR Kerr S Kilty CG Serjeant GR. Haematological response to parvovirus B19 infection in sickle-cell disease. Lancet. (2001) 358:1779–80. 10.1016/S0140-6736(01)06807-6 11734237

[13] Abraham M Rudraraju R Kannangai R George K Cherian T Daniel D. A pilot study on the seroprevalence of parvovirus B19 infection. Indian J Med Res. (2002) 115:139–43. 12239835

[14] Watson-Williams EJ. Folic acid deficiency in sickle-cell anaemia. E Afr Med J. (1962) 39:213–21. 14005324

[15] Rabb LM Grandison Y Mason K Hayes RJ Serjeant BE Serjeant GR. A trial of folate supplementation in children with homozygous sickle cell disease. Br J Haematol. (1983) 54:589–94. 10.1111/j.1365-2141.1983.tb02138.x 6347243

[16] Readett DRJ Serjeant BE Serjeant GR. Hurricane Gilbert anaemia. Lancet. (1989) 2:101–2. 10.1016/S0140-6736(89)90337-1 2567840

[17] Intervention Program for Nutritional Anemia and Haemoglobinoathies amongst some Primitive Tribal Populations of India. A National Multicentric Study of ICMR 2000-2005. ICMR New Delhi (2010).

[18] Mohanty D Mukherjee MB Colah RB Wadia M Ghosh K Chottray GP. Iron deficiency anaemia in sickle cell disorders in India. Indian J Med Res. (2008) 127:366–9. 18577791

[19] Morgan AG Serjeant GR. Renal function in patients over 40 with homozygous sickle cell disease. Br Med J. (1981) 282:1181–3. 10.1136/bmj.282.6271.1181 6788125 PMC1505256

[20] Hayes RJ Beckford M Grandison Y Mason K Serjeant BE Serjeant GR. The haematology of steady state homozygous sickle cell disease. Frequency distributions, variation with age and sex, longitudinal observations. Br J Haematol. (1985) 59:369–82. 10.1111/j.1365-2141.1985.tb03002.x 2578806

[21] Rogers DW Clarke JM Cupidore L Ramlal AM Sparke BR Serjeant GR. Early deaths in Jamaican children with sickle cell disease. Br Med J. (1978) 1:1515–6. 10.1136/bmj.1.6126.1515 656779 PMC1605039

[22] Emond AM Collis R Darvill D Higgs DR Maude GH Serjeant GR. Acute splenic sequestration in homozygous sickle cell disease: natural history and management. J Pediatr. (1985) 107:201–6. 10.1016/S0022-3476(85)80125-6 4020541

[23] Badaloo AV Singhal A Forrester TE Serjeant GR Jackson AA. The effect of splenectomy for hypersplenism on whole body protein turnover, resting metabolic rate and growth in sickle cell disease. Eur J Clin Nutr. (1996) 50:672–5. 8909934

[24] Stevens MCG Padwick M Serjeant GR. Observations on the natural history of dactylitis in homozygous sickle cell disease. Clin Pediatr. (1981) 20:311–7. 10.1177/000992288102000501 7226681

[25] Serjeant GR De Ceulaer C Lethbridge R Morris JS Singhal A Thomas PW. The painful crisis of homozygous sickle cell disease - clinical features. Br J Haematol. (1994) 87:586–91. 10.1111/j.1365-2141.1994.tb08317.x 7993801

[26] Baum KF Dunn DT Maude GH Serjeant GR. The painful crisis of homozygous sickle cell disease: a study of risk factors. Arch Int Med. (1987) 147:1231–4. 10.1001/archinte.147.7.1231 3606281

[27] Platt OS Thorington BD Brambilla DJ Milner PF Rosse WF Vichinsky E. Pain in sickle cell disease. Rates and risk factors. N Engl J Med. (1991) 325:11–6. 10.1056/NEJM199107043250103 1710777

[28] Ware ME Hambleton I Ochaya I Serjeant GR. Sickle cell painful crises in Jamaica: a day care approach to management. Br J Haematol. (1999) 104:93–6. 10.1046/j.1365-2141.1999.01160.x 10027718

[29] Benjamin LJ Swinson GI Nagel RL. Sickle cell anemia day hospital: an approach for the management of uncomplicated painful crises. Blood. (2000) 95:1130–7. 10.1182/blood.V95.4.1130.003k03a_1130_1136 10666181

[30] Knight-Madden J Serjeant GR. Invasive pneumococcal disease in homozygous sickle cell disease: Jamaican experience 1973-1997. J Pediatr. (2001) 138:65–70. 10.1067/mpd.2001.109709 11148514

[31] John AB Ramlal A Jackson H Maude GH Waight-Sharma A Serjeant GR. Prevention of pneumococcal infection in children with homozygous sickle cell disease. Br Med J. (1984) 288:1567–70. 10.1136/bmj.288.6430.1567 6426646 PMC1441216

[32] Gaston MH Verter JI Woods G Pegelow C Kelleher J Presbury G. Prophylaxis with oral penicillin in children with sickle cell anemia. N Engl J Med. (1986) 314:1593–9. 10.1056/NEJM198606193142501 3086721

[33] Pearson HA Spencer RP Cornelius EA. Functional asplenia in sickle-cell anemia. N Engl J Med. (1969) 281:923–6. 10.1056/NEJM196910232811703 5811425

[34] Pearson HA McIntosh S Ritchey AK Lobel JS Rooks Y Johnston D. Developmental aspects of splenic function in sickle cell diseases. Blood. (1979) 53:359–65. 10.1182/blood.V53.3.358.bloodjournal533358 760858

[35] Serjeant GR. Irreversibly sickled cells and splenomegaly in sickle-cell anaemia. Br J Haematol. (1970) 19:635–41. 10.1111/j.1365-2141.1970.tb01647.x 5481507

[36] Padmos MA Roberts GT Sackey K Kulozik A Bail S Morris JS. Two different forms of homozygous sickle cell disease occur in Saudi Arabia. Br J Haematol. (1991) 79:93–8. 10.1111/j.1365-2141.1991.tb08013.x 1716963

[37] Casper JT Koethe S Rodey GE Thatcher LGA. new method for studying splenic reticuloendothelial dysfunction in sickle cell disease patients and its clinical application: a brief report. Blood. (1976) 47:183–8. 10.1182/blood.V47.2.183.183 1244917

[38] Al-Awamy B Wilson WA Pearson HA. Splenic function in sickle cell disease in the Eastern Province of Saudi Arabia. J Pediatr. (1984) 104:714–7. 10.1016/S0022-3476(84)80950-6 6201602

[39] Serjeant BE Hambleton IR Serjeant GR. Retained splenic function in an Indian population with homozygous sickle cell disease may have important clinical significance. Indian J Community Med. (2021) 46. 10.4103/ijcm.IJCM_1054_20 PMC8729266 35068741

[40] Rogers DW Serjeant BE Serjeant GR. Early rise in 'pitted' red cell counts as a guide to susceptibility to infection in childhood sickle cell anaemia. Arch Dis Child. (1982) 57:338–42. 10.1136/adc.57.5.338 7092288 PMC1627578

[41] Wong WY Powars DR Chan L Hiti A Johnson C Overturf G. Polysaccharide encapsulated bacterial infection in sickle cell anemia: a thirty year epidemiologic experience. Am J Hematol. (1992) 39:176–82. 10.1002/ajh.2830390305 1546714

[42] Wright J Thomas P Serjeant GR. Septicemia caused by salmonella infection an overlooked complication of sickle cell disease. J Pediatr. (1997) 130:394–9. 10.1016/S0022-3476(97)70201-4 9063414

[43] Patel J Patel B Serjeant GR. The bone pain crisis of sickle cell disease and malaria: Observations from Gujarat, India. Indian J Community Med. (2017) 42:167–9. 10.4103/ijcm.IJCM_334_16 28852282 PMC5561696

[44] Cumming V King L Fraser R Serjeant G Reid M. Venous incompetence, poverty and lactate dehydrogenase in Jamaica are important predictors of leg ulceration in sickle cell anaemia. Br J Haematol. (2008) 142:119–25. 10.1111/j.1365-2141.2008.07115.x 18477043

[45] Serjeant GR Hambleton I. Priapism in homozygous sickle cell disease: a 40 year study of the natural history. W Ind Med J. (2015) 64:175–80. 10.7727/wimj.2014.119 26426165 PMC4763886

[46] Jain DL Apte M Colah R Sarathi V Desai S Gokhale A. Efficacy of fixed low dose hydroxyurea in Indian children with sickle cell anemia: a single centre experience. Indian Pediatr. (2013) 50:929–33. 10.1007/s13312-013-0264-0 23798623

[47] Jain DL Sarathi V Desai S Bhatnagar M Lodha A. Low fixed-dose hydroxyurea in severely affected Indian children with sickle cell disease. Hemoglobin. (2012) 36:323–32. 10.3109/03630269.2012.697948 22734586

[48] Jain D Tokalwar R Upadhye D Colah R Serjeant GR. Homozygous sickle cell disease in Central India & Jamaica: A comparison of newborn screening cohorts. Indian J Med Res. (2020) 151:326–32. 10.4103/ijmr.IJMR_1946_18 32461396 PMC7371056

